# The contribution of cognitive reserve in explaining the dual-task walking performance in iNPH patients: comparison with other cognitive, functional, and socio-demographic variables

**DOI:** 10.1007/s40520-024-02829-0

**Published:** 2024-09-11

**Authors:** Elodie Piche, Stephane Armand, Gilles Allali, Frederic Assal

**Affiliations:** 1 Université Côte d’Azur, LAMHESS, Nice, France; 2 Université Côte d’Azur, Centre Hospitalier Universitaire de Nice, Clinique Gériatrique du Cerveau et du Mouvement, Nice, France; 3 Laboratory of Kinesiology, University Geneva Hospitals and University of Geneva, Geneva, Switzerland; 4 Leenaards Memory Center, Lausanne University Hospitals and University of Lausanne, Lausanne, Switzerland; 5 Division of Neurology, Department of Clinical Neurosciences, University Geneva Hospitals, Geneva, Switzerland

**Keywords:** Dual-task walking, Idiopathic normal pressure hydrocephalus, Cognitive reserve

## Abstract

**Background:**

Idiopathic normal pressure hydrocephalus (iNPH) is a prevalent neurological disorder, but its diagnosis remains challenging. Dual-task (DT) walking performance is a reliable indicator of iNPH but less is known about the role of cognitive reserve (CR) in predicting DT walking performance.

**Aims:**

The objective of this study was to evaluate the contribution of CR on DT walking in healthy controls (HC) and in iNPH patients (iNPH-P).

**Methods:**

68 iNPH-P (77.2 +/- 6.7 years old) and 28 HC (74.5 +/- 5.7 years old) were evaluated on their single-task walking (*Vsimple*) and on 4 DT walking (walking and counting or counting backwards, naming animals, naming words beginning with the letter P) (*Vcount*,* VcountB*,* Vanimals and Vletter* respectively). The contribution of CR on the different DT walking speeds was compared between HC and iNPH-P. In iNPH-P, the contribution of CR on the walking speeds was compared with regard to other cognitive, functional, and socio-demographic variables.

**Results:**

Simple linear regression demonstrated a moderate influence of CR on single and DT walking speed in iNPH-P (β > 0.3, *p* < .001) but not in HC where the relation was not significant. In iNPH-P, results showed that CR played a major role in explaining each of the single and DT walking speeds with NPH-scale.

**Conclusion:**

As CR could be improved through the life cycle, these results support the idea of developing and supporting physical activity programs that will enrich social, physical, and cognitive resources to protect against age-related functional decline, especially in iNPH-P patients where the age-related deficits are greater.

## Background

Idiopathic normal pressure hydrocephalus (iNPH) is a prevalent reversible neurological disorder characterized by impaired locomotion, cognition, and urinary control with ventriculomegaly at brain imaging [1]. However, the diagnosis of iNPH remains challenging. Indeed, iNPH symptoms are not specific and can be found in other neurological disorders, such as Parkinson’s disease or Alzheimer’s disease (e.g. iNPH mimics) [2, 3]. There is also a lack of iNPH-specific diagnostic markers and several guidelines exist based on different criteria [4]. Therefore, depending on the guideline used, the prevalence of iNPH varies, which likely leads underdiagnosis [5]. The accuracy of diagnosing patients with iNPH can also be debated, as less than 60% of iNPH patients showed improvement after treatment [6]. This directly impacts the cost-effectiveness of the shunt intervention. However, recent studies found that adding dual-task walking to the cerebrospinal fluid tap test (CSF) may help to identify iNPH from its mimics [7, 8]. Indeed, iNPH patients were differentiated from the mimics by a greater improvement of their dual-task gait parameters (walking speed, stride length, step width, and stance duration) after the CSF tap test while, in single-task walking, gait improvements after the CSF tap test were similar between iNPH patients and iNPH mimics [8]. The difference in DT between the mimics and the patients could possibly be due to the lack of cognitive resources in iNPH patients which may resulted in greater impairments in DT before the CSF tap test. However, after the removal of the iNPH symptoms with the CSF tap test, it is conceivable that the DT walking would be largely improved compared to the mimics.

Indeed, dual-tasking consists of the simultaneous execution of two tasks, generally cognitive and/or motor tasks of different levels of complexity (for example, counting while walking). Under DT conditions, performance of one or both tasks can vary relative to single conditions because of competing demands. They are due to insufficient attentional resources or an inability to share these limited attentional resources between two or more concurrent tasks, thus causing a decline in performance [9, 10]. Resource competition has been found to be particularly pronounced in older adults with limited cognitive resources like in iNPH, and the DT paradigm has emerged as a promising marker for predicting cognitive impairment [11–13], fall risk [13–15] or frailty status [16–18] in older adults. However, the dual-task performance is not well understood as it could depend on many different factors such as age, frailty or other aspects of cognitive, physical/functional, psychosocial or social–demographic aging [18, 19].

The cognitive reserve (CR) strongly relies on the idea that there can be individual differences in how tasks are processed that can allow some people to cope better than others with brain changes in general and aging in particular [20]. CR suggests that the brain actively attempts to cope with brain damage by using pre-existing cognitive processes or by enlisting compensatory processes [21]. A recent study showed that CR played a role of moderator in the relationship between gait velocity under single and dual-task conditions and incident mobility impairment [22]. In other words, slower single-task walking and dual-task walking velocity predicted incident mobility impairment only among those individuals with low CR [22]. It is, thus, necessary to better understand which specific factors could influence dual-task walking performance in iNPH population as dual-task walking can help therapeutic decisions for neurosurgical shunt placement.

This study aims to evaluate the contribution of CR on DT walking in healthy controls (HC) and iNPH patients (iNPH-P). Our first hypothesis was that the relation between CR and dual-task walking is higher in iNPH-P compared to HC. The second hypothesis was that a high level of CR measured by the level of education is positively correlated with a high dual-task walking speed in each speed condition and that this variable contributes more importantly to the dual-task walking speed than other specific clinical measures in iNPH-P.

## Materials and methods

### Participants

Sixty-eight iNPH patients (iNPH-P) and 28 healthy controls (HC) were included in this retrospective study from the NPH cohort recruited at a tertiary referral hospital between March 2017 and February 2020 according to a previously described protocol [7]. Briefly, iNPH-P patients were recruited when they met the iNPH consensus guideline criteria [1]. The patients’ gait was analyzed twice, before and then 24 h after a CSF tapping of 40 ml according to a standardized protocol [7, 23]. Exclusion criteria included: acute medical illness in the past three months, orthopedic or rheumatologic disorders interfering with gait, patients receiving CSF tapping in the 3 months preceding the assessment, a change in the treatment between the two gait assessments, unable to walk a minimum of 15 m without a walking aid and not able to perform the dual-task evaluation (walking while backward counting, naming animals or letter). Gait disorders, cognitive impairment, and urinary disturbance were graded using the iNPH grading scale [24]. The iNPH grading scale was used to separately evaluate the severity of each of the three disorders. The score of each domain ranges from 0 to 4 with higher scores indicating worse symptoms. The study protocol was approved by the Institutional Review Board of the Geneva University Hospital (Protocol 09-160R). All subjects gave informed consent according to the ethical standards set forth in the declaration of Helsinki (1983).

### Dual-task gait protocol

Patients were asked to walk at their self-selected speed on a 10-m walkway in single and in four randomized dual-task conditions: forward counting from 1 (*Vcount*); backward counting from 50 (*VcountB*); phonemic verbal fluency (enumerating words starting by letter p) (*Vletter*); and categorical verbal fluency (generating animals’ name) (*Vanimals*). A standardized instruction for the dual-tasks was given by the physical therapist: to walk and to perform the cognitive task at the best of their capacity without prioritization. Mean value and coefficient of variability of spatiotemporal gait parameters (that include walking speed) were computed based on the measurement of the heel marker trajectories on a 10 meters’ distance with an optoelectronic system including 12 cameras (Oqus7+, Qualisys Sweden). The gait assessment was conducted before and 24 h after CSF tap test pre-CSF tap test, which is described in the Geneva’s protocol [7].

### Cognitive reserve

CR could be estimated using different proxies such as the level of education, premorbid intelligence quotient (IQ) or vocabulary size measured by the number of words pronounced in 1mn [21, 25]. In this study, CR was measured with the level of education measured in three categories (1, 2 and 3, respectively up to 9 years of education or elementary school, 9–12 years or apprentices, more than 12 years, or higher education) in HC and iNPH-P and expressed in years in iNPH-P.

### Covariates

Then, the influence of CR was compared with other variables such as age and gender as socio-demographic variables, the total score of MMSE (Mini mental State Examination) as an indication of the cognitive function, NPH scale, disease duration in months, the age-related white matter changes (ARWMC) and global health status score (GHS) as functional/physical variables. Specifically, the NPH grading scale allows quantifying the NPH symptoms in gait, cognition, and miction using a Likert scale from 0 to 12 [24]. The ARWMC was used to quantify the burden of white matter disease in all the brain and its sub-regions [26]. Then, the GHS (0–10) summarizes the presence of the following comorbidities: diabetes, chronic heart failure, arthritis, hypertension, depression, stroke, PD (Parkinson’s disease), chronic obstructive pulmonary disease, angina, and myocardial infarction [27].

### Statistics

Descriptive statistics presented socio-demographic variables and all the dual-task walking speed conditions (*Vsimple*,* Vcount*,* VcountB*,* Vanimals*,* Vletter*) in iNPH-P and HC participants (Table 1). Student’s t-test was applied, or Mann-Whitney when the variable deviated from normality, to compare each variable between the two groups. Then, a simple linear regression was applied between each speed (*Vsimple*,* Vcount*,* VcountB*,* Vanimals and Vletter*) and CR measured with the level of education in category (0–3). A comparison of the relation between the influence of CR on the different speeds between NPH-P and NPH-C was assessed with a two-way ANOVA with each speed as a dependent variable and the level of education (1–3) and groups (iNPH-P, HC) as fixed factors. To go further on iNPH-P group only, a Spearman’s rho correlation or Pearson was applied when appropriated between the 5 walking speed conditions (*Vsimple*,* Vcount*,* VcountB*,* Vanimals*,* Vletter*) and age, gender, CR, MMSE, NPH-scale, disease duration, ARWMC and GHS separately. To determine which of these 8 variables can better influence the walking speed in single and DT walking conditions, multiple linear regression analysis (MLRA) was applied to each of the walking speed condition as dependent variables with the covariates mentioned above which are age, gender, CR, MMSE, NPH-scale, disease duration, ARWMC and GHS. The MLRA was used with the stepwise method that removed at each step the least useful predictor. For each of the simple or multiple linear regression, normality, or skewness around − 2 and 2 was checked as well as heteroscedasticity and collinearity (VIF < 5). The independence of errors was also checked with a Durbin-Watson value between 1.5 and 2.5. All statistical analyses were performed with JASP (version 0.16.6, JASP Team (2023), The Netherlands).

## Results

Sixty-eight iNPH-P (mean aged: 77.2 +/- 6.7 years old) and 28 HC (mean aged: 74.5 +/- 5.7 years old) were recruited for this study (Table 1).

**Table 1:** Socio-demographic and clinical measures for iNPH-P (normal pressure Hydrocephaly-Patient) and HC (normal pressure Hydrocephaly-Control) patients (mean (SD))

	iNPH-P (*n* = 68)	HC (*n* = 28)	*p*-value	Effect size (ES)
Age (years)	77.2 (6.7)	74.5 (5.7)	0.031a	-0.282
Male, n (%)	40 (58.8)	7 (25)	Na	Na
Female, n (%)	28 (41,2)	21 (75)	Na	Na
MMSE (/30)	24.5 (3.7)	27.8 (1.3)	< .001a	0.590
Educational level (0-1-2-3)	2.3 (0.7)	2.6 (0.6)	Na	Na
GHS	1.7 (1.1)	Na	Na	Na
ARWMC	6.8 (4.6)	Na	Na	Na
NPH-scale (0–12)	4.9 (1.5)	Na	Na	Na
TUG time (s)	20.9 (10.3)	10.9 (2.1)	< .001a	-0.861
10 m-walk test (s)	15.8 (7)	10 (1.5)	< .001a	-0.747
Vsimple (m/s)	0.78 (0.2)	1.24 (0.15)	< 0.001	0.359
Vcount (m/s)	0.74 (0.3)	1.21 (0.16)	< 0.001	0.352
VcountB(m/s)	0.64 (0.24)	1.11 (0.19)	< 0.001	0.359
Vanimals (m/s)	0.58 (0.25)	0.99 (0.19)	< 0.001	0.327
Vletter (m/s)	0.54 (0.22)	0.96 (0.21)	< 0.001	0.346

a: Mann-Whitney test for non-normal values

*Note* For the Student t-test, effect size is given by Cohen’s d. For the Mann-Whitney test, effect size is given by the rank biserial correlation

*Abbreviations* MMSE: Mini Mental State Examination; GHS: Global Health Status score; ARWMC: Age-Related White Matter Changes; NPH-scale: Normal Pressure Hydrocephalus-scale; TUG: Timed up and go

Concerning the influence of CR measured with the level of education in categories on each of the different walking conditions, the simple linear regression was only significant in iNPH-P compared to HC where the regression was not significant (Table 2). There was also a moderate positive association between CR and the different walking conditions in iNPH-P as shown by the standardized coefficient in the linear regression (β > 0.4, *p* < .001) (Table 2). Thus, the influence of CR on the different walking speeds seemed to be applicable only in iNPH-P.

**Table 2:** Simple linear regression with the different walking speed (Vsimple, Vcount, VcountB, Vanimals, Vletter) as dependent variables and CR as a covariate in iNPH-P and HC

Model	*R*²		ANOVA		intercept		β	
	iHPN-*P*	iHPN-C	iHPN-*P*	iHPN-C	iHPN-*P*	iHPN-C	iHPN-*P*	iHPN-C
Vsimple	0.164	0.015	F(1,67) = 12.920, *p* < .001	F(1,27) = 0.407, *p* = .529	0.455	1.322	0.405	-0.124
Vcount	0.152	< 0.001	F(1,67) = 11.828, *p* = .001	F(1,27) = 0.005, *p* = .944	0.736	1.222	0.390	-0.014
VcountB	0.192	0.003	F(1,67) = 15.659, *p* < .001	F(1,27) = 0.068, *p* = .796	0.296	1.144	0.438	-0.051
Vanimals	0.190	0.002	F(1,67) = 15.484, *p* < .001	F(1,27) = 0.049, *p* = .827	0.210	1.032	0.436	-0.043
Vletter	0.212	0.014	F(1,67) = 17.766, *p* < .001	F(1,27) = 0.365, *p* = .551	0.199	0.860	0.461	0.118

**Fig. 1:**
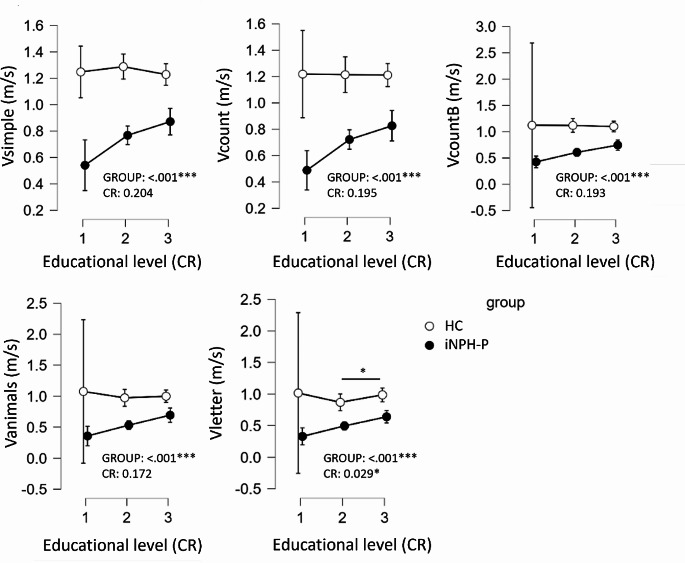
ANOVA results with errors bars (confidence intervals 95%) comparing walking speeds (Vsimple, Vcount, VcountB, Vanimals and Vletter) between GROUPS (HC and iNPH-P) through their different educational levels (CR). Each p-value for the independent variable GROUP and CR is shown. *p < .05, **p < .01, ***p < .001

Also, ANOVA results highlighted that the different walking speeds were not significantly different among the different CR levels (1, 2 or 3 based on their educational level) (CR: *p* > .05) except for Vletter (*p* = .029) where the walking speed significantly increase with a higher level of CR (Fig. 1). However, all the walking speeds were significantly higher in HC compared to iNPH-P (GROUP: *p* < .001) (Fig. 1). No significant differences were found in the effect CR (1, 2, 3) *group (iNPH-P, HC) (*p* > .05).

When focusing only on iNPH-P, correlation coefficients highlighted that only NPH-scale was significantly correlated to all the walking speeds with a moderate strength (R²>0.3, *p* < .01) (Table 3). Age was also negatively correlated to the walking speed but with a weak strength (R²<0.3, *p* < .05) (Table 3). The total score of MMSE was correlated to the walking speeds except with *Vcount* and *Vletter* when GHS was significantly correlated with *VcountB*, *Vanimals* and *Vletter* but not with *Vsimple* nor *Vcount*. Gender and disease duration were not significantly correlated to the different walking speeds (Table 3). As mentioned before, CR was moderately correlated with the walking speeds in iNPH-P (Table 2) and (Table 3).

**Table 3:** For iNPH-P group, correlation between each dual-task walking speeds and the 8 clinical measures. Pearson or Spearman correlation was applied when appropriate

	Vsimple	Vcount	VcountB	Vanimals	Vletter
Age *a*	-0.28*	-0.307*	-0.272*	-0.264*	-0.299*
Gender *b*	-0.184	-0.193	-0.164	-0.149	-0.107
Disease duration *a*	0.063	0.045	0.103	0.204	0.184
Educational level (CR) *a*	0.424***	0.403***	0.418***	0.429***	0.450***
MMSE *a*	0.282*	0.195	0.356**	0.247*	0.226
NPH scale *b*	-0.391***	-0.406***	-0.461***	-0.379**	-0.316**
GHS *b*	-0.231	-0.214	-0.318**	-0.300*	-0.292*
ARWMC *a*	-0.173	-0.168	-0.244*	-0.253*	-0.139

*Note* (a) Pearson correlation; (b) Spearman’s rho coefficient correlation *p < .05 ; **p < .01 ; ***p < .001

To explore which variables could play a major role in explaining the physical performance through single and dual-task walking speeds, multiple linear regression analysis (MLRA) was used. The MLRA for each of the different walking speeds *(Vsimple*,* Vcount*,* VcountB*,* Vanimals*,* Vletter)* was built with *age*,* gender*,* disease duration*,* educational level (CR)*,* NPH scale*,* GHS and ARWMC* as variables. Each model was significant and explained more than 35% of the variability of the different speeds with the best model for VcountB (F (4.63) = 12.880, *p* < .001) (Table 4). Results identified CR, NPH scale, and age as the main contributors to all the different walking speeds with an averaged slope in each model of 0.366, -0.302, -0.297 respectively. The positive slope coefficient for CR means that an increase in CR is associated with an increase in walking speed and inversely for NPH scale and age as the slope is negative. GHS appeared to be also a contributor for *VcountB*,* Vanimals and Vletter* with a slope coefficient of -0.226, -0.206, and − 0.216 respectively, *p* < .05 (Table 4).

**Table 4:** Multiple linear regression model (stepwise) summary for all the dual-task walking speeds (Vsimple, Vcount, VcountB, Vanimals, Vletter) in iNPH-P. Most contributive parameters are in bold

Model	*R*² (%)	ANOVA	intercept	Variables in the best model	β	*p*
Vsimple	36.1	F (3.64) = 12.076, *p* < .001	4.821	**Educational level (CR)**	**0.386**	< 0.001
				**NPH scale**	**-0.311**	0.003
				age	-0.274	0008
Vcount	37.1	F (3.64) = 12.603, *p* < .001	4.906	**Educational level (CR)**	**0.364**	< 0.001
				**NPH scale**	**-0.328**	0.002
				age	-0.298	0.004
VcountB	45	F (4.63) = 12.880, *p* < .001	5.327	**NPH scale**	**-0.372**	< 0.001
				**Educational level (CR)**	**0.331**	0.001
				Age	-0.292	0.003
				GHS	-0.226	0.022
Vanimals	39	F (4.63) = 10.075, *p* < .001	4.352	**Educational level (CR)**	**0.359**	< 0.001
				NPH scale	-0.288	0.006
				Age	-0.288	0.005
				GHS	-0.206	0.046
Vletter	39.8	F (4.63) = 10.424, *p* < .001	4.632	**Educational level (CR)**	**0.392**	< 0.001
				**Age**	**-0.331**	0.001
				NPH scale	-0.215	0.034
				GHS	-0.216	0.035

Included covariates in all models were age, gender, disease duration, education level, MMSE, NPH-scale, GHS, total ARWMC

## Discussion

The present study aimed to evaluate the contribution of CR on DT walking in healthy controls (HC) and iNPH patients (iNPH-P). The first hypothesis of this study was partially validated as the simple linear regression highlighted a moderate positive association between CR and the different walking speeds as shown by the standardized coefficient in the linear regression (β > 0.4, *p* < .001) (Table 2) but only applicable in iNPH-P and not in HC. Then, the multiple linear regression analysis highlighted the role of education, above and beyond all the other considered factors, as the best predictor of dual-task walking speed variability in the specific iNPH population which confirmed the second hypothesis.

First, a moderate positive association between CR and the different walking speeds was shown by the standardized coefficient in the simple linear regression (Table 2). However, these results were applicable only in iNPH-P but not in HC (Table 2). These findings were not in line with previous studies that found significant interaction effects of CR with walking speed under ST (b = 0.09, 95% CI [0.01;0.17], z = 2.30, *p* = .02) and DT (b = 0.10, 95% CI [0.02;0.17], z = 2.55, *p* = .01) conditions in normal aging [22]. Many studies also found that single gait speed increases with the highest level of education in normal aging [28, 29]. The non-significant results in HC could be explained by the low repartition of the HC participants in the category 1 of the level of education. Indeed, as Fig. 1 highlighted, there was a large standard deviation in the first category of the level of education, explained by only two HC participants included in this group. Thus, by increasing the number of participants in this category, the correlation between CR and mobility performance (*Vsimple*,* Vcount*,* VcountB*,* Vanimals and Vletter*) would have been greater and similar to previous studies. However, the present findings reinforce the idea that the level of education contributes to cognitive reserve as a compensatory factor of aging declines [21, 30]. Holtzer et al. [31] found that CR was associated with a lower risk of developing incident mobility impairment (odds ratio (OR) = 0.819, 0.67–0.98, *p* = .038 (unadjusted)), which illustrates the protective role of CR against age-related declines. Education may increase the predisposition for greater physical and mental stimulation during the entire lifetime which, in turn, may generally contribute to greater CR and, more specifically, to higher flexibility in dual-task contexts, as shown here with a higher walking speed in DT and elsewhere [32, 33]. The protective effect of CR against adverse health outcomes appeared to be greater in the population with the greatest cognitive and functional impairments, thus the iNPH-P (see MMSE score, TUG time, and 10 m-walk test in Table 1). These results are interesting as the recommendation proposed by O’Brien’s study could be all the more interesting in vulnerable populations like iNPH-P [22]. Indeed, O’Brien et al. [22] supported the idea that the moderating role of CR in the relationship between gait velocity and incident mobility impairment highlights the importance of building and maintaining one’s reserve capabilities in order to protect against age-related functional decline. Research has shown that CR can increase throughout the life span in response to enriching social, physical, and cognitive activities [34], suggesting that healthcare providers and those involved in caring for older adults should recommend participation in such activities to support healthy aging. It could be an interesting recommendation in the specific iNPH-P population where the effect of CR on mobility performance seemed to be higher.

Concerning iNPH-P specifically, the multiple linear regression analysis highlighted that the role of CR measured by the level of education was the best predictor with NPH-scale of single and dual-task walking speed above and beyond all the other considered factors (Table 4). This is in line with a study that has shown through an MLRA that education was the best predictor of dual-task cost variability in healthy aging above and beyond all the other considered factors which were age, general cognitive abilities measured with the MOCA score, and secondary task efficiency [33]. Interestingly, the other important variables that explain walking speed in iNPH-P were NPH scale, age and GHS. The NPH scale considered three domains of NPH which are gait impairments, urinary disturbances, and cognitive impairment [24]. As the NPH-scale is closely related to gait performance, its correlation with single and dual-task walking is not so surprising. What is surprising is that, despite the close link of NPH scale with gait, CR was the most correlated with walking speed in each model (Table 4) except in *VcountB*. Age was the third contributive variable to single and dual-task walking speeds with a negative slope suggesting that single and dual-task walking speeds decrease with aging. Many studies shared these results where single and dual-task walking performances were strongly influenced by age [18]. GHS, which is a global score that considers various aspects of health status such as diabetes, chronic heart failure, arthritis, hypertension, depression, stroke, chronic obstructive pulmonary disease, angina, or myocardial infarction, was also a major contributive variable to *VcountB*,* Vanimals and Vletter* with a mean negative slope of -0.216 in all models (Table 4). The negative slope means that slower gait speed is associated with a higher GHS-score and, thus, a higher number of comorbidities. No study has demonstrated the effect of this precise GHS-score on walking performance, but it has already been shown that low gait speed was associated with a higher number of medications (OR = 4.28, 95% CI [1.63, 11.2]), and a higher number of depressive symptoms (OR = 1.31, 95% CI [1.09, 1.58]) [35]. Busch et al. [28] also found that slower gait speed was associated with cardiovascular disease. This is of special importance as cerebrovascular conditions, a major consequence of cardiovascular disease, are often reported in iNPH [36, 37]. Gait and especially dual-task walking requires a high level of cognitive capacities such as sustaining attention and executive function [38]. As patients with comorbidities had poorer performance in executive functions and memory compared to controls [39, 40], it could be hypothesized that this cognitive limitation leads to a decline in walking performance when the cognitive capacity is exceeded during high attention-demanding task in accordance with the capacity limitations hypothesis [41] which could explain the correlation obtained in MLRA. However, with respect to these different clinical variables, CR remained the most contributive variable of the single and dual-task walking conditions.

### Limitations

Finally, this study replicated previous findings on the positive contribution of CR on mobility performance usually measured by walking speed. Also, the results of this study highlighted new variables that highly contributed to single and dual-task walking speed such as NPH-scale, age or GHS (Table 4) in a population with an important number of iNPH patients. Noted that radiological measures for iNPH such as DESH or the radscale were not analyzed in this study but it should be interesting to take into account [4]. However, several limitations need to be discussed. As mentioned before, the unequal repartition of the iNPH-P and HC participants through the three categories of the level of education makes the interpretation and the comparison between the two groups difficult. Also, it is important to notice that the level of education was measured continuously in iNPH-P which is the most common way to assess the level of education while, in HC, only the categorical variable was accessible which could influence the correlation results and bring a ceiling effect. Concerning the MLRA, the guideline ratio for a linear regression to detect reasonable-size effects with reasonable power is to have 1 covariate for at least 10 participants [42]. Based on this recommendation, it was expected to have 80 participants as the MLRA included 8 covariates but only 68 participants were included in this study. Thus, the MLRA’s result may not be generalized to other new data as including too many covariates relative to the number of participants can lead to overfitting, where the model fits the noise in the data rather than the underlying patterns. Thus, it could be interesting to test the model on a new set of data to check for replicability and overfitting. In this study, only direct correlations were tested but future studies should explore the moderation between each covariate rather than direct effects. Analyzing moderator effects could offer a greater contrast in gait speed changes across conditions, especially in iNPH population to better characterize the responder to CSF tap test and improve the diagnosis. It is also important to notice that the patients unable to perform the dual task were not include in this study which may limit the representativeness of this specific iNPH population.

## Conclusions

In summary, this study found that single and dual-task performance was strongly related to CR in iNPH-P but not in HC. Indeed, the relation between CR measured with the level of education and different walking speeds (*Vsimple*,* Vcount*,* VcountB*,* Vanimals*,* Vletter*) seemed to be different between the two groups. CR was also more closely related to single and dual-task walking speeds than other clinical variables such as *age*,* gender*,* NPH scale*,* MMSE score*,* disease duration*,* and comorbidity burden measured with the GHS and ARWMC* in the specific iNPH-P population. As CR could be improved through the life cycle [34], these results support the idea of developing and supporting physical activity programs or other activities that will enrich social, physical and cognitive resources in order to protect against age-related functional decline, especially in the specific iNPH-P population.

## Data Availability

No datasets were generated or analysed during the current study.
